# Unveiling the Metabolic Fingerprint of Occupational Exposure in Ceramic Manufactory Workers

**DOI:** 10.3390/toxics14010056

**Published:** 2026-01-07

**Authors:** Michele De Rosa, Silvia Canepari, Giovanna Tranfo, Ottavia Giampaoli, Adriano Patriarca, Agnieszka Smolinska, Federico Marini, Lorenzo Massimi, Fabio Sciubba, Mariangela Spagnoli

**Affiliations:** 1 Department of Chemistry, Sapienza University of Rome, Piazzale Aldo Moro 5, 00185 Rome, Italy; michele.derosa@uniroma1.it (M.D.R.); adriano.patriarca@uniroma1.it (A.P.); federico.marini@uniroma1.it (F.M.); 2 NMR-Based Metabolomics Laboratory, Sapienza University of Rome, Piazzale Aldo Moro 5, 00185 Rome, Italy; ottaviagiampaoli@gmail.com (O.G.); fabio.sciubba@uniroma1.it (F.S.); 3 Department of Environmental Biology, Sapienza University of Rome, Piazzale Aldo Moro 5, 00185 Rome, Italy; silvia.canepari@uniroma1.it (S.C.); l.massimi@uniroma1.it (L.M.); 4 Department of Medicine, Epidemiology, Environmental and Occupational Hygiene, National Institute for Insurance against Accidents at Work (INAIL), Via Fontana Candida 1, 00078 Monte Porzio Catone, Italy; g.tranfo@inail.it; 5 Faculty of Health, Medicine and Life Sciences, Department of Pharmacology and Toxicology, Maastricht University, 6200 MD Maastricht, The Netherlands; a.smolinska@maastrichtuniversity.nl; 6 School of Nutrition & Translational Research in Metabolism (NUTRIM), Maastricht University, 6211 LK Maastricht, The Netherlands; 7 Research Center for Applied Sciences to the safeguard of Environment and Cultural Heritage (CIABC), Sapienza University of Rome, Piazzale Aldo Moro 5, 00185 Rome, Italy

**Keywords:** NMR spectroscopy, metabolomics, multivariate statistical analysis, occupational exposure, workers biomonitoring, urinary profiles

## Abstract

In this study, for the first time urinary NMR-based metabolomics was applied to investigate the physiological alterations associated with occupational exposure in ceramic manufacturing workers. Multivariate analysis revealed a distinctive metabolic signature with exposure, characterized by a depletion of both aliphatic and aromatic amino acids and a concomitant accumulation of branched-chain amino acid catabolites. Alterations in tricarboxylic acid (TCA) cycle intermediates, including citrate and succinate, suggest an involvement of mitochondrial energy metabolism, reflecting adaptive responses to oxidative stress and increased protein turnover. Notably, glycine levels were found increased, consistent with its central role in antioxidant defense and xenobiotic detoxification. Furthermore, changes in urinary host–microbiome co-metabolites, such as 4-hydroxyphenylacetate and phenylacetylglycine, indicate the potential modulation of gut microbial activity in response to occupational exposure. While limited by the small cohort, this study demonstrates the feasibility of NMR-based urinary metabolomics for the non-invasive biomonitoring of workers and suggests its potential as a useful tool for detecting subtle metabolic perturbations associated with complex occupational exposures.

## 1. Introduction

The Italian artistic ceramics sector represents a prominent component of the broader Made in Italy ecosystem, supported by a centuries-old artisan tradition and the innovation of contemporary design. Italian ceramics encompass various styles, including majolica, terracotta, and porcelain, each with distinctive characteristics and traditions. Several regions in Italy, like Deruta, Vietri sul Mare, and Caltagirone, are particularly famous for their unique ceramic traditions and craftsmanship. In 2022, the entire Italian ceramics industry, including tiles and slabs, sanitaryware, porcelain and tableware, refractory materials, technical ceramics, and heavy clay products, contributed to a turnover of approximately €8.7 billion, with 259 companies and more than 26,000 direct employees [1]. Within this, the ceramic tiles segment alone generated almost €7.2 billion, €6 billion of which came from exports, which accounted for over 80% of the turnover. These figures underscore the critical economic as well as cultural importance of artistic ceramic production in Italy’s domestic and global markets. However, this artisanal excellence comes with significant occupational chemical risks.

Traditional artistic ceramic manufacturing involves a multistage process, from clay preparation to decoration, glazing, and firing, each of which presents specific chemical hazards for workers. Initially, clay materials, composed of hydrated aluminium silicates, are mixed with water and sometimes additives such as grog, talc, perlite, and metal oxides. This phase generates dust containing free crystalline silica, a Group 1 carcinogen, according to IARC classification [2,3], which is strongly associated with silicosis, kaolinosis, and other pneumoconiosis following chronic inhalation [4]. The shaped pieces, produced by hand modelling, wheel throwing, or casting, are then dried and moved to the bisque firing stage, where they are heated at temperatures around 900–1000 °C to harden the structure. This step oxidizes organic matter in the clay and can release carbon monoxide, sulphur oxides, and volatile fluorides, depending on the raw materials used [5]. After bisque firing, the objects proceed to the decoration phase, which is central in artistic ceramics. Here, metal oxide pigments, stains, and luster compounds are applied by brushing, dipping, or spraying. These materials often contain toxic metals such as lead, cadmium, chromium (VI), cobalt, and nickel. The glazing process follows, involving the application of a vitreous coating composed of silica, fluxes (e.g., lead oxides, alkaline/alkaline earth oxides), and modifiers such as boric oxide or alumina. Glaze powders are typically very fine and highly dispersible, creating high exposure potential during weighing, mixing, and handling. The final glaze firing stage subjects the decorated pieces to a second firing at 950–1100 °C. This step vitrifies the glaze, forming a durable surface, but may also lead to the volatilization of heavy metals and toxic gases, including lead fumes, antimony, cadmium, SO_2_, NO_x_, and HF depending on glaze composition [6]. In view of what has been stated, workers are exposed to a complex mixture of hazardous substances, making it difficult to assess potential health effects induced by occupational exposure through more conventional biological monitoring strategies.

Metabolomics, defined as “the quantitative measurement of the dynamic multiparametric metabolic response of living systems to pathophysiological stimuli or genetic modification” [7], aims to provide a measure of the low molecular weight (<1500 Da) metabolites present in a given biological matrix. In recent decades, the characterization of the quali-quantitative composition of the metabolome has gained an increasingly central role in system biology, given that the metabolome is the level mostly related to the phenotype expressed by an individual, and therefore its alterations represent the organism’s ways of adapting to the external environment.

Among the available analytical platforms, Nuclear Magnetic Resonance (NMR) spectroscopy represents the election technique for small compound structure elucidation, allowing for simultaneous quali-quantitative analyses of real complex samples with minimum sample pretreatment [8]. The present work is the first case in which an NMR-base metabolomic approach is applied to characterize urinary profiles of workers employed in the ceramic manufacturing sector with the aim of revealing potential correlations between occupational exposure and alterations in basal metabolism.

## 2. Materials and Methods

### 2.1. Study Design

Eighteen workers employed in two different ceramic manufacturing companies located in the same geographical area in Italy were enrolled, and a one-spot urine sample was collected for each subject. The selected companies were comparable in terms of the manufactured products and employed processes. On the basis of this homogeneity and taking into account the small number of employees of each company, all workers were placed in a single group (hereafter named as Workers). Regarding the tasks carried out by each worker, it can be said that almost all of them were employed in decoration. At the same time, twenty-nine healthy volunteers (hereafter named as CTRL) were included in the study as a control group, and a one-spot urine sample was collected analogously to the workers. Subjects included in the control group were recruited externally from the companies, and their occupation was known to rule out workplace exposure to other hazards. The absence of pathologies that could significantly impact metabolism (e.g., diabetes, cancer, or other metabolic diseases), drug consumption, and main lifestyle habits of each subject included in the study were assessed through the administration of specific surveys. Demographic characteristics of both workers and healthy volunteers are resumed in Table 1.

All urine samples were collected in the middle of the working week and at the beginning of the working shift after overnight starvation and immediately stored at −20 °C until analysis. All experiments were conducted according to the Declaration of Helsinki and followed the International Code of Ethics for Occupational Health Professionals, published by the International Committee of Occupational Health (ICOH). The information gathered was used as aggregate data referring to the whole group, with no risk of individual identification. This study was approved by the Ethical Committee “CET Lazio Area 2”, study protocol: “BRIC ID 52”, protocol ID:113.24 CET2 ptv. Written informed consent was obtained from all subjects involved.

### 2.2. Sample Preparation

All urine samples were pretreated following a previously described protocol for biofluid metabolomics analysis [9] adapted according to [10]. In more detail, each sample was centrifuged at 4 °C for 15 min at 11,000 rpm to remove any precipitate or cellular debris. Subsequently, 400 µL of the supernatant was added to 200 µL of buffer solution (PBS 200 mM, pH = 7) with 60 µL of a solution of the internal standard 3-(trimethylsilyl) propionic-2,2,3,3-d4 acid sodium salt (TSP) in D_2_O.

### 2.3. NMR Spectroscopy Experimental Settings

A JEOL JNM-ECZR spectrometer (JEOL Ltd., Tokyo, Japan), equipped with a magnet operating at 14.09 Tesla and 600.17 MHz for the ^1^H working frequency, was used for recording all NMR spectra. The following setting parameters were employed: temperature of 298 K, 64 k points and 64 scans, spectral width at 9.03 kHz (15 ppm), presaturation pulse length of 2.00 s for water signal suppression, relaxation delay of 5.72 s, and acquisition time of 5.81 s. To allow for compound identification, bidimensional experiments were also carried out (Figure S2). In particular, ^1^H-^1^H TOCSY experiments were carried out at 298 K using a spectral width of 15 ppm for both direct and indirect dimensions with an 8 k × 128 data point matrix, a 3.00 s repetition time and 80 scans; 80.00 ms were used for the spinlock sequence. The ^1^H-^13^C HSQC analyses were performed at 9.03 KHz (15 ppm) for the proton dimension and 30 KHz (200 ppm) for the carbon dimension, setting 8 k × 128 data point matrix and with 2 s of repetition delay and 96 scans. ^1^H-^13^C HMBC pulse sequences were acquired employing spectral widths of 9.03 KHz (15 ppm) for the proton dimension and 37.5 KHz (250 ppm) for the carbon dimension, a data matrix of 8 k × 128 points, a repetition delay of 2 s, 96 scans, and a long-range coupling constant ^1^H-^13^C of 8 Hz.

### 2.4. Spectral Preprocessing

All spectra were preprocessed using the ACD Labs software v.12.0 (Advanced Chemistry Development, Inc., 8 King Street East, Toronto, ON, Canada). The preprocessing step consisted of the application of an exponential window function (LB = 0.3 Hz), the application of Fourier Transform, manual phasing, and baseline correction, applying the baseline correction FID reconstruction (BCFR) procedure as reported elsewhere [10]. The chemical shift scale of each spectrum was referenced by setting the TSP singlet to 0.00 ppm, and preprocessed raw profiles were subjected to alignment employing the icoshift algorithm [11]. Adaptive Intelligent Binning (AIB) was then applied as reported elsewhere [12] after a further baseline refinement. The AIB algorithm adjusts bin widths based on spectral features to align them with peak boundaries, reducing the risk of peak fragmentation and improving quantification accuracy. In particular, for the case in question the following input parameters were used: study region from 0.25 to 10.00 ppm, dark region removed from 4.60 to 6.00 ppm including the residual signals of water and urea. Finally, the optimization of the quality parameter for choosing the width of the bins took place according to the indications provided by the authors who proposed the method [12]. Given the high correlation existing between variables obtained from omics platforms, the data were analyzed through multivariate statistical analysis techniques performed on the data matrix following PQN normalization, log-transformation, and autoscaling (Supplementary Table S2). Metabolite assignment in urinary NMR spectra was performed using a combination of approaches. Firstly, metabolite annotation was guided by the inspection of 1D ^1^H-NMR spectra and supported by the Chenomx NMR Suite 10.1 software (Chenomx Inc., Edmonton, AB, Canada), and then preliminary identifications were cross-checked against online freely available databases and previously published literature [13,14,15,16,17,18]. Finally, to ensure robust and reliable metabolite identification, two-dimensional NMR experiments were systematically employed for confirmation. In particular, TOCSY, HSQC, and HMBC spectra were acquired to verify correlations, resolve signal overlap in congested spectral regions, and confirm metabolite-specific connectivity patterns. A list of identified metabolites with relative resonances and chemical shift values are reported in Supplementary Table S1.

### 2.5. Statistical Analysis

Unsupervised Random Forest (URF) was applied as an explorative technique [19,20]. Fifty performed iterations, five samples in last leaf, and number of trees equal to fifteen hundred were chosen as tuning parameters. URF was used as a valid alternative to the more common PCA, being more robust in the presence of outliers and capable of accounting for both linear and non-linear effects maintaining model interpretability. With the aim of identifying those variations in the urinary metabolome that are potentially correlated with occupational exposure, a Partial Least Square discriminant analysis (PLS-LDA) model was built. A repeated double cross-validation [21] was chosen as the validation procedure, and model performance was evaluated by the following figures of merit: sensitivity, specificity, accuracy, and correct classification rate. To account for potential confounding effects of age, sex, and smoking status, we implemented a covariate-adjusted partial least squares discriminant analysis (PLS-DA) using a residualization strategy. Specifically, for each metabolite feature, a linear regression model was fitted within the training data of each cross-validation fold, with the metabolite intensity as the dependent variable and the selected covariates as independent variables. Smoking status and sex were encoded using dummy variables where appropriate. The residuals from these models, representing metabolite variation not explained by the covariates, were then used to construct a covariate-adjusted feature matrix.

PLS-DA models were subsequently trained on the residualized metabolite matrix and evaluated on the corresponding residualized test data, ensuring that covariate adjustment was performed exclusively within each training fold to prevent information leakage. Model performance was assessed using a repeated double cross-validation scheme, with classification metrics reported from the outer validation loop. The statistical significance of class separation was further evaluated using permutation testing, in which class labels were randomly permuted to generate a null distribution of performance metrics.

This approach enables an assessment of whether group discrimination persists after the removal of variance attributable to major confounders while maintaining strict separation between training and test data. Residualization-based covariate adjustment was preferred over the inclusion of covariates as predictors to ensure that group discrimination reflected metabolite-specific variation rather than direct effects of demographic or lifestyle factors. The classification performance of the covariate-adjusted PLS-DA models is summarized below. Statistics were carried out employing MATLAB 2024b (Natick, MA, USA: The MathWorks Inc.) and in-house written functions.

## 3. Results

**Figure 1:**
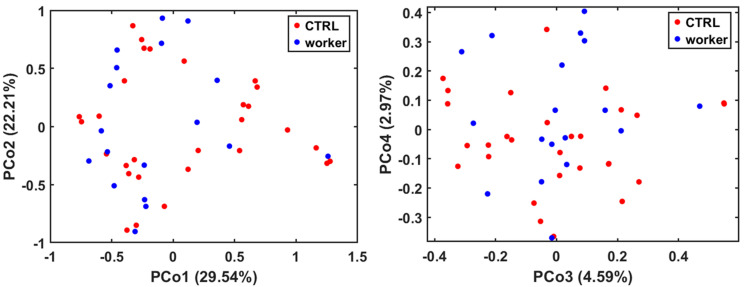
Score plots obtained from Unsupervised Random Forest analysis.

A representative ^1^H-NMR spectrum of urine sample with assigned metabolite resonances is reported in Supplementary Figure S1. URF analysis was applied with the purpose of assessing the occurrence of the spontaneous clustering of data as well as the presence of samples exhibiting anomalous behavior. As observable from score plots reported in Figure 1, no isolated groups were found, nor were any peculiar profiles that were extremely different from the majority of samples identified.

**Figure 2:**
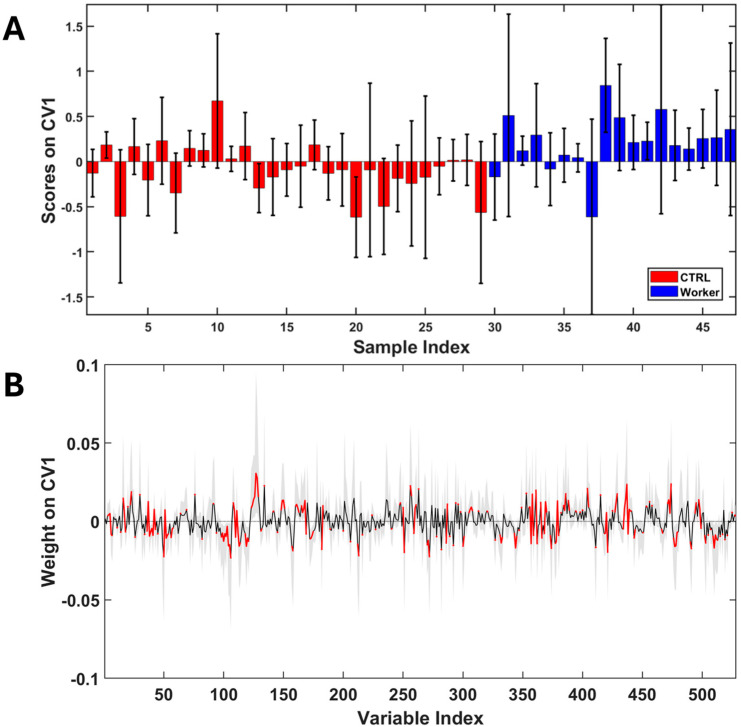
(**A**) PLS-LDA scores along the first canonical variate. (**B**) PLS-LDA weights along the first canonical variate. The black line represents the mean weight profile, and the grey shaded area indicates the 95% confidence interval for each variable. Segments highlighted in red correspond to variables whose confidence interval bounds do not cross the threshold of 0 and that are considered significant for discrimination.

An important aspect to underline is that metabolomic analysis can be prone to the presence of confounding factors such as the diet, age, smoke, drugs intake, life habits, and gender of enrolled subjects. In order to verify how much some of these spurious factors impact the dissimilarity between samples, the same score plots reported previously have been represented with different color codes in Supplementary Figure S3. What emerges from this representation is that confounding factors not even considered plausible impact sample grouping. This last statement does not entirely rule out the presence of confounders that could affect the analysis; it merely proves that these do not represent the main source of variance characterizing the data. Therefore, what can be deduced from this first analysis is that inter-individual biological variability remains the major source of variance present in the dataset. Therefore, on the basis of this assumption of homogeneity and taking into account the small sample size available, it was decided not to exclude any subjects from the subsequent analyses. After the first explorative analysis and with the aim of focusing only on the differences induced on metabolome by occupational exposure, a PLS-LDA discriminant model was built (Figure 2). The model performances were evaluated through a double cross validation procedure obtaining following validation parameters in discriminating workers from healthy volunteers: 70 ± 5% (accuracy); 60 ± 8% (sensitivity); 76 ± 6% (specificity); 68 ± 5% (correct classification rate).

As reported previously, the covariate adjustment of the PLS-DA model was also performed. Adjustment for individual covariates resulted in modest changes in performance. Specifically, adjustment for smoking yielded an accuracy of 69 ± 5% with increased sensitivity (73 ± 7%) and reduced specificity (63 ± 8%), whereas adjustment for sex or age led to accuracies of 66 ± 5% and 64 ± 5%, respectively. When all three covariates (age, sex, and smoking) were adjusted for simultaneously, classification performance decreased further but remained above chance, with an accuracy of 63 ± 4%, a sensitivity of 66 ± 6%, and a specificity of 58 ± 8%. Permutation testing confirmed that class separation remained statistically significant across models, indicating that the observed discrimination was unlikely to arise from random label assignment. Together, these results demonstrate that group-related metabolic differences persist after the removal of variance attributable to major confounding factors, although the overall signal strength is moderate.

The analysis of weights along the first canonical variate (Figure 2B) revealed a significant increase in urinary concentrations of 3-hydroxyisobutyrate (3-HIBA), erythro-2,3-dihydroxybutyrate (2,3-DHB), 2-hydroxyisobutyrate (2-HIBA), glycine, 4-hydroxyphenyl acetate (4-HPAA), and phenylacetylglycine (PAG) in workers. In contrast, levels of valine, alanine, citrate, succinate, tyrosine, tryptophan, hippurate, and formate decrease with exposure.

## 4. Discussion

The first aspect that stands out from the results presented points to a set of biochemical changes that may be consistent with an imbalance in mitochondrial energetic metabolism with exposure. This hypothesis is supported by an alteration of key tricarboxylic cycle intermediates (succinate and citrate). It is known from the literature that exposure to xenobiotics such as heavy metals could interfere with physiological mitochondrial function and energy metabolism [22]. Indeed, heavy metals can interfere with both cells’ ability to maintain a stable redox balance and the electron transport chain (ETC) [23,24,25]. ETC represents a crucial part of mitochondrial energy production, and its impairment can lead to a dysregulation of ATP synthesis and potentially to cell damage. Exposure risk to heavy metals is in line with the occupational context considered, as highlighted above, given the use by workers of materials such as glazes and paints. Furthermore, exposure to silica dust, a common component of ceramic manufacturing, has also been observed to impact mitochondrial metabolism in humans, leading to various cellular dysfunctions [26,27]. Specifically, silica dust can induce mitochondrial swelling, membrane disruption, and apoptosis, ultimately affecting ATP production and cellular respiration. This damage is linked to the generation of reactive oxygen species (ROS), mediators of oxidative stress conditions, and to the activation of inflammatory pathways, known to underlie the development of silicosis and respiratory diseases resulting from silica exposure [27,28,29].

Another interesting aspect concerns the fairly widespread decrease in urinary levels of both aliphatic and aromatic amino acids found in workers. This alteration, combined with the concomitant increase in the concentration of some branched-chain amino acid catabolites (2-HIBA, 3-HIBA, 2,3-DHB), allows us to advance two different hypotheses. On the one hand, it is plausible that an effect of increasing protein turnover is observed. In this case, the free amino acids would be preserved for the repair of protein structures damaged by the oxidative stress mechanisms. These mechanisms, as already anticipated, are notoriously connected to exposure to particulates containing metals and silica dusts, as in the case under examination [30,31,32,33]. Alternatively, it should not be overlooked that amino acid metabolism is closely linked to mitochondrial activity. Indeed, almost all twenty proteinogenic amino acids are degraded and synthesized in the mitochondria. A recent and very detailed review [34] highlighted the crosstalk between amino acid metabolism and mitochondrial homeostasis. Among the many aspects covered in the cited work, it is discussed how amino acids can also constitute important substrates for the tricarboxylic acid cycle (TCA). In brief, amino acid catabolites can feed the TCA cycle as well as TCA intermediates being able to serve as precursors for amino acid synthesis [35]. These processes became especially important during conditions of nutrient or energy stress, where amino acids can be mobilized for energy. In light of this, the observed depletion of amino acids such as valine, alanine, tyrosine, and tryptophan could reflect enhanced amino acid catabolism in response to mitochondrial dysfunction and increased energy demand following imbalance in the organism redox state. Regarding the amino acid glycine, this is the only one to exhibit completely opposite behavior to all the others. This behavior is likely due to this compound’s central role in antioxidant and defense mechanisms such as glutathione synthesis or xenobiotic phase II detoxification reactions.

The metabolites 4-HPAA and PAG are also intermediates in amino acid catabolism. However, both compounds are best described as host–microbiome co-metabolites rather than direct one-step products of host amino acid catabolism. Multiple gut bacteria convert dietary and host-derived tyrosine and phenylalanine into smaller aromatic acids (including 4-HPAA and phenylacetate) via specific microbial pathways; these microbially produced aromatic acids are then taken up by the host and further processed by hepatic conjugation reactions before renal excretion [36,37]. Several commensal taxa can oxidize tyrosine to 4-HPAA in the gut, and here 4-HPAA can then be absorbed systemically and exert biological effects. Its urinary abundance therefore reflects microbial production as much as the host supply of tyrosine. Thus, an increased urinary 4-HPAA may indicate an increased microbial conversion of tyrosine (e.g., dysbiosis or blooming of specific taxa), greater dietary tyrosine availability, or altered host absorption/clearance, or any combination of the three [38]. For PAG, the pathway is similarly bifurcated: gut microbes convert phenylalanine into phenylacetate (PAA), and the host then activates phenylacetate to phenylacetyl-CoA and conjugates it with glycine through glycine N-acyltransferase to yield PAG, which is eliminated in urine. Several studies have shown marked reductions in circulating PAG and PAG-related metabolites when the microbiota is suppressed, demonstrating the microbial origin of the PAA precursor. Therefore, urinary PAG reports on both microbial phenylacetate production and the host’s glycine-conjugation capacity [39,40]. Host factors modulate the final urinary excretion. Interindividual variation in hepatic glycine N-acyltransferase activity, glycine availability, liver function, and renal clearance can all change how much microbial phenylacetate ends up as urinary PAG.

Overall, this hypothesis-generating study provides an exploratory overview of metabolic differences associated with occupational exposure in ceramic workers. While the observed metabolomic patterns are consistent with previously described effects of metal and silica exposure on mitochondrial metabolism, amino acid turnover, and host–microbiome co-metabolism, the cross-sectional design and lack of direct functional measurements limit causal interpretation. Future studies integrating metabolomics with targeted biomarkers of oxidative stress, mitochondrial function, dietary assessment, and microbiome profiling will be necessary to validate and further elucidate the biological significance of our findings.

## 5. Conclusions

The presented work represents, to the best of our knowledge, the first pilot study investigating the urinary metabolic profiles of workers employed in artistic ceramic manufacturing. The investigated sector plays a significant role in the national economy, and the complexity of its exposure scenario makes it an excellent context for the application of innovative biomonitoring strategies. Our results revealed a metabolic signature consistent with mitochondrial energetic imbalance and oxidative stress in accordance with literature data. Alterations in tricarboxylic acid cycle intermediates, together with a generalized depletion of amino acids and a concomitant rise in branched-chain amino acid catabolites, suggest an adaptive metabolic shift aimed at sustaining cellular energy requirements under stress conditions. The observed perturbations let us speculate that exposure to heavy metals and silica dust, both common components of ceramic manufacturing, may interfere with mitochondrial function, redox homeostasis, and amino acid metabolism. In addition, changes in the urinary excretion of host–microbiome co-metabolites, such as 4-HPAA and PAG, highlight a possible involvement of gut microbial metabolism in the systemic response to occupational exposure. These findings could emphasize the interconnectedness of mitochondrial dysfunction, amino acid catabolism, and gut microbial activity as key elements in the biological response to xenobiotic-induced stress.

Nevertheless, as a pilot and hypothesis-generating investigation, this study presents some limitations that should be carefully considered when interpreting the results. First, the relatively small cohort size limits the generalizability of the observed metabolic patterns. In addition, the use of a single spot urine sample per participant may be influenced by transient factors making the hypotheses advanced biologically plausible but preliminary. Future perspectives should therefore consider a larger cohort of enrolled subjects to strengthen the study’s statistical power and confirm the observed metabolic trends. Moreover, integrating NMR metabolomics with other analytical platforms and with environmental monitoring data could certainly provide a more comprehensive understanding of the molecular mechanisms underlying exposure-related effects. Overall, our results underline the value of metabolomics as an early and non-invasive approach for detecting metabolic alterations possibly linked to complex occupational exposures, paving the way for more advanced and holistic biomonitoring strategies aimed at improving workers’ health protection.

## Figures and Tables

**Table 1:** Characteristics of enrolled subjects.

	Age (mean ± SD)	Males (N)	Females (N)	Smokers (N)
workers	48 ± 10	7	11	6
CTRLS	55 ± 7	8	21	4

## Data Availability

The original contributions presented in this study are included in the article/Supplementary Materials. Further inquiries can be directed to the corresponding author.

## Supplementary Materials

The following supporting information can be downloaded at: https://www.mdpi.com/article/10.3390/toxics14010056/s1, Figure S1: ^1^H-NMR spectrum of typical urine sample; Figure S2: 2D-NMR experiments performed on urine samples; Figure S3: Score plots from URF analysis. Table S1: ^1^H chemical shifts of metabolite signals in urines. (bs: broad singlet; d: doublet; dd: doublet of doublets; m: multiplet; q: quadruplet; s: singlet; t: triplet). Significant metabolites for discrimination between exposed workers and controls are reported in bold and their percentage variation between groups is also calculated; Supplementary Table S2: raw data produced in the study.
