# Supplementary material for: Unveiling the Metabolic Fingerprint of Occupational Exposure in Ceramic Manufactory Workers
Source: Toxics. 2026 Jan 7;14(1):56. doi: 10.3390/toxics14010056 (PMC12845652; doi:10.3390/toxics14010056)
Supplement: Supplementary file 1 [file toxics-14-00056-s001.zip › toxics-4014472-supplementary.pdf]

Supplementary

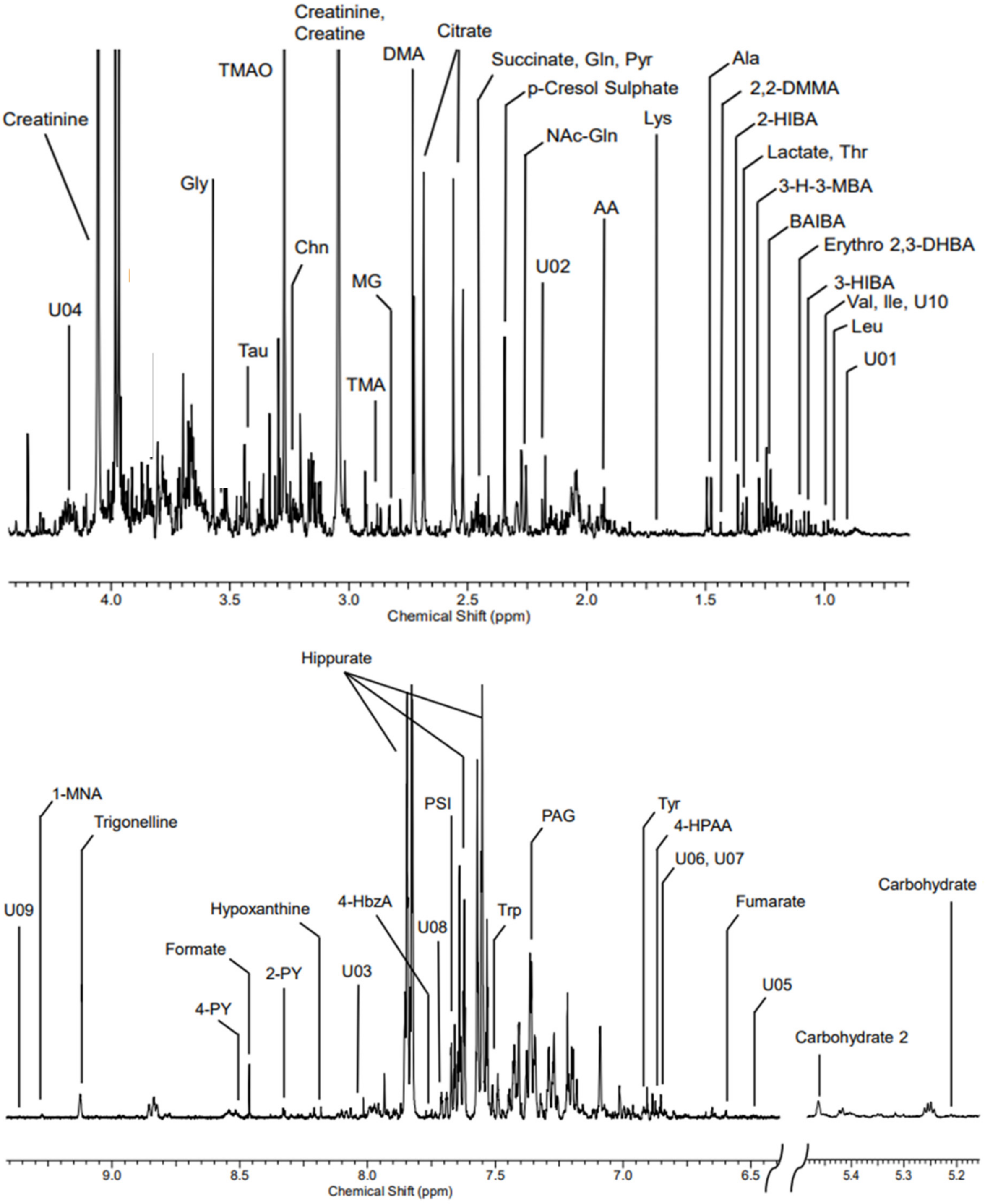

Figure S1. ^{1}H-NMR spectrum of typical urine sample in regions **(up)** 0.5 - 4.4 ppm and **(bottom)** 5.2 – 9.5 ppm. Resonances are referenced to TSP singlet at 0.00 ppm (not shown). The dark regions between 4.5 – 5.5 ppm and 5.4 – 6.5 ppm correspond to the resonances of water and urea respectively. List of abbreviations: leucine (Leu), valine (Val), isoleucine (Ile), 3-hydroxyisobutyrate (3-HIBA), erythro-2,3-dihydroxubutyrate (erythron 2,3-DHB), 3-aminoisobutanoic acid (BAIBA), 3-hydroxy-3-methylbutyrate (3-H-3-MBA), threonine (Thr), 2-hydroxyisobutyrate (2-HIBA), 2,2-dimethylmalonate (2,2-DMMA), alanine (Ala), lysine (Lys), acetate (AA), N-acetylglutamine (NAc-Gln), glutamine (Gln), pyruvate (Pyr), dimethylamine (DMA), methylguanidine (MG), trimethylamine (TMA), choline (Chn), trimethylamine-N-oxide (TMAO), taurine (Tau), glycine (Gly), 4-hydroxyphenylaetate (4-HPAA), tyrosine (Tyr), phenylacetylglycine (PAG), tryptophane (Trp), pseudouridine

(PSI), 4-hydroxybenzoate (4-HBz), N-methyl-2-pyridone-5-carboxamide (2-PY), N-methyl-4-pyridone-3-carboxamide (4-PY), 1-methylnicotinamide (1-MNA), unknown compound (U).

Molecule	Structure	^{1}H δ (ppm)	Assignment	Multiplicity	^{13}C δ (ppm)	Percentage variation
**1. Valine (Val)**	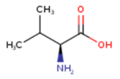	**0.99**<br>**1.04**	**CH3**<br>**CH3'**	**d**<br>**d**		**-5%**
2. Isoleucine (Ile)	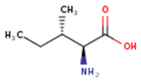	1.01	β-CH3	d		
3. Leucine (Leu)	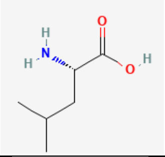	0.95	CH3, CH3'	dd		
**4. 3-Hydroxyisobutyric acid (3-HIBA)**	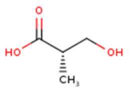	**1.08**<br>**2.49**<br>**3.53**<br>**3.70**	**CH3**<br>**α-CH**<br>**β-CH**<br>**β-CH'**	**d**	**16.00**	**+5%**
**5. Erythro-2,3-dihydroxybutyric acid (Erythro-2,3-DHBA)**	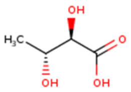	**1.11**<br>**4.12**	**CH3**<br>**α-CH**	**d**		**+30%**
6. β-aminoisobutyric acid (BAIBA)	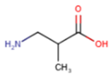	1.20	CH3	D		
7. 3-Hydroxy-3-methylbutyric acid (3-H-3-MBA)	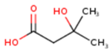	1.27<br>2.37	CH3, CH3'<br>CH2	s<br>s	30.90	
8. Lactic acid (LA)	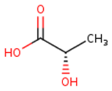	1.33<br>4.11	CH3<br>α-CH	d<br>q	22.90	
9. Threonine (Thr)	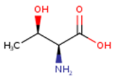	1.34<br>3.60<br>4.27	CH3<br>α-CH<br>β-CH	d	22.90	
**10. 2-Hydroxyisobutyric acid (2-HIBA)**	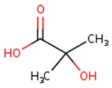	**1.36**	**CH3, CH3'**	**s**	**29.5**	**+33%**
11. Dimethylmalonic acid (DMMA)	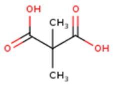	1.44	CH3, CH3'	s		
**12. Alanine (Ala)**	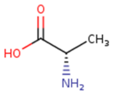	**1.48**<br>**3.78**	**CH3**<br>**α-CH**	**d**<br>**q**	**19.15**	**-37%**

13. Lysine (Lys)	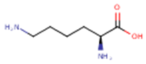	1.48<br>1.71<br>1.89<br>3.02<br>3.74	CH2<br>CH2<br>CH2<br>CH2<br>CH	m<br>m<br>m<br>m<br>m		
14. Acetic acid (AA)	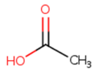	1.93	CH3	s	26.30	
15. N-acetylglutamine (NAcGln)	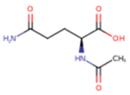	1.94<br>2.16<br>2.27<br>4.19<br>7.97	β-CH<br>β'-CH<br>γ-CH2<br>α-CH<br>NH	m<br>m<br>m<br>m<br>bd	34.7	
16. Glutamine (Gln)	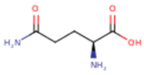	2.13<br>2.46<br>3.78	β -CH2<br>γ-CH2<br>α-CH	m<br>m<br>dd	33.83	
17. p-Cresol sulfate (p-CrS)	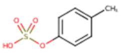	2.35<br>7.20<br>7.29	CH3<br>2,4-CH<br>3,5-CH	s	22.5	
18. Pyruvic acid (Pyr)	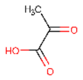	2.53	CH3	S		
**19. Succinic Acid (SA)**	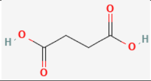	**2.41**	**CH2, CH2'**	**s**		**-9%**
**20. Citric acid (CA)**	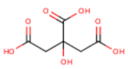	**2.54**<br>**2.69**	**α,β-CH2**<br>**α',β'-CH2**	**d**<br>**d**	**48.04**<br>**48.04**	**-24%**
21. Dimethylamine (DMA)	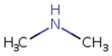	2.73	CH3, CH3'	s	37.9	
22. Methylguanidine (MG)	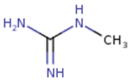	2.83	CH3	bs	30.10	
23. Trimethylamine (TMA)	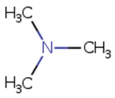	2.89	CH3, CH3', CH3''	s	45.80	
24. Creatine (Crt)	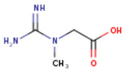	3.05<br>3.94	CH3<br>CH2	s<br>s	39.80<br>56.85	
25. Creatinine (Crtn)	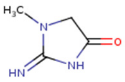	3.05<br>4.06	CH3<br>CH2	s<br>s	32.96<br>59.32	
26. Choline (Chn)	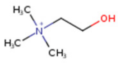	3.22	N-CH3,CH3',CH3''	s	57.20	

27. Taurine (Tau)	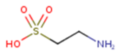	3.27<br>3.43	CH2<br>CH2	t<br>t	50.50<br>38.66	
28. Trimethylamine-N-Oxide (TMAO)	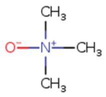	3.27	CH3, CH3’, CH3’’	s	62.08	
**29. Glycine (Gly)**	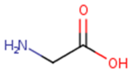	**3.57**	**CH2**	**s**	**44.5**	**+15%**
**30. Formic acid**	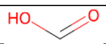	**8.45**	**CH**	**s**	**175.00**	**-14%**
**31. 4-Hydroxyphenylacetic acid (4-HPAA)**	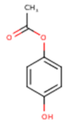	**6.86**<br>**7.16**	**3,5-CH**<br>**2,6-CH**	**dd**<br>**dd**		**+20%**
**32. Tyrosine (Tyr)**	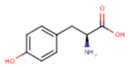	**6.90**<br>**7.19**	**3,5-CH**<br>**2,6-CH**	**dd**<br>**dd**		**-20%**
33. 4-Hydroxybenzoic acid (4-HBzA)	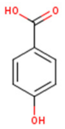	6.97<br>7.76	3,5-CH<br>2,6-CH	d<br>d	132.18	
**34. Tryptophan (Trp)**	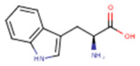	**7.20**<br>**7.27?**<br>**7.29?**<br>**7.50**<br>**7.70**	**3-CH**<br>**2-CH**<br><br>**5-CH**<br>**4-CH**	**pd**<br>**pd**	**115.23**	**-5%**
**35. Phenylacetylglycine (PAG)**	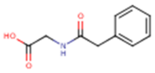	**7.34-7.43**	**2-5 CH**	**m**	**131.5**	**+50%**
**36. Hippuric acid (HippA)**	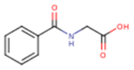	**3.97**<br>**7.55**<br>**7.64**<br>**7.83**	**CH2**<br>**3,5-CH**<br>**4-CH**<br>**2,6-CH**	**d**<br>**m**<br>**m**<br>**m**	**46.4**<br>**131.71**<br>**135.18**<br>**130.09**	**-18%**
37. Pseudouridine (PSI)	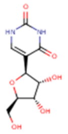	7.67	CH	s	144.5	
38. Hypoxanthine (Hyp)	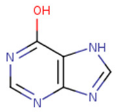	8.19<br>8.21	2-CH<br>7-CH	s<br>s		
39. N1-Methyl-2-pyridone-5-carboxamide (2PY)	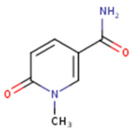	3.64<br>6.67?<br>7.98?<br>8.33	N-CH3<br>3-CH<br>4-CH<br>6-CH	s<br>d<br>d<br>dd	182.12	

40. N1-Methyl-2-pyridone-3-carboxamide (2PY)	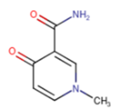	8.50	6-CH	dd		
41. Formic acid (FA)	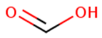	8.46	CH	s	169.23	
42. Trigonelline (Trig)	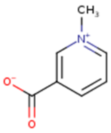	4.34<br>8.08<br>8.84<br>9.12	N-CH3<br>5-CH<br>4,6-CH<br>2-CH	s<br>m<br>m<br>s	 <br><br>148.5	
43. 1-Methylnicotinamide (1-MNA)	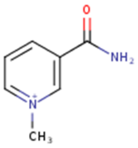	4.44<br>8.17<br>8.89<br>8.96<br>9.28	N-CH3<br>5-CH<br>4-CH<br>6-CH<br>2-CH	s<br>t<br>d<br>d<br>s		

Table S1. ^1H chemical shifts of metabolite signals in urines. (bs: broad singlet; d: doublet; dd: doublet of doublets; m: multiplet; q: quadruplet; s: singlet; t: triplet). Significant metabolites for discrimination between exposed workers and controls are reported in bold and their percentage variation between groups is also calculated.

^{1}H-^{1}H TOCSY

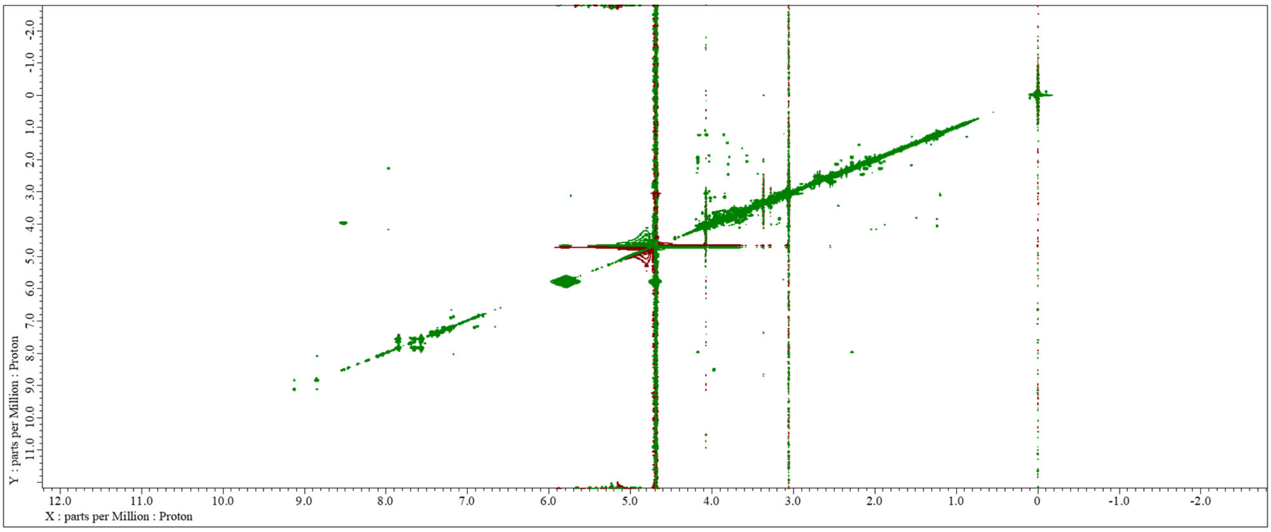

^{1}H-^{13}C HSQC

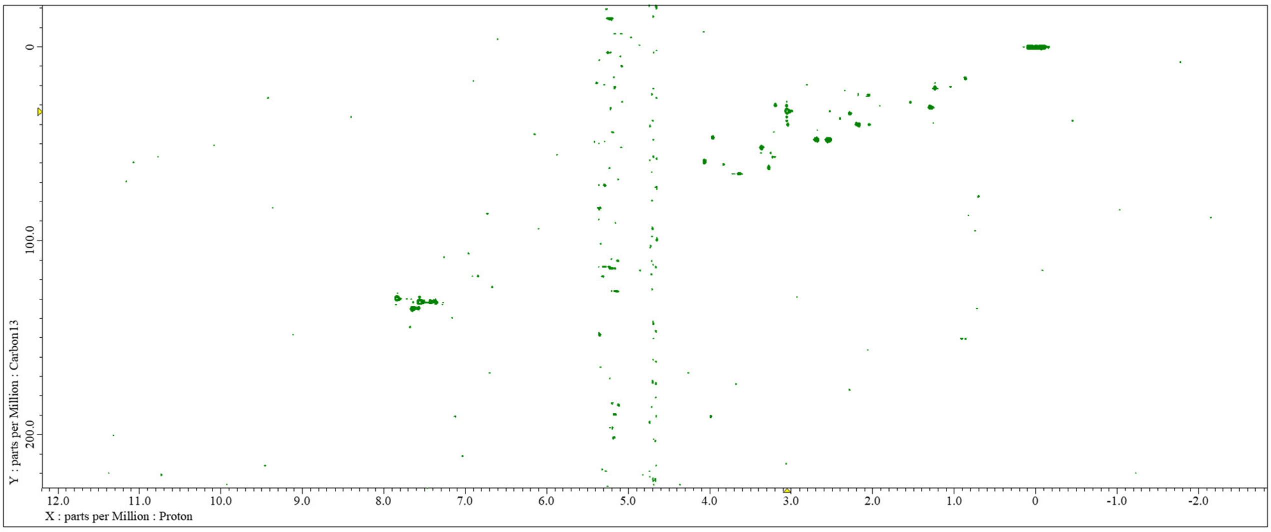

^{1}H-^{13}C HMBC

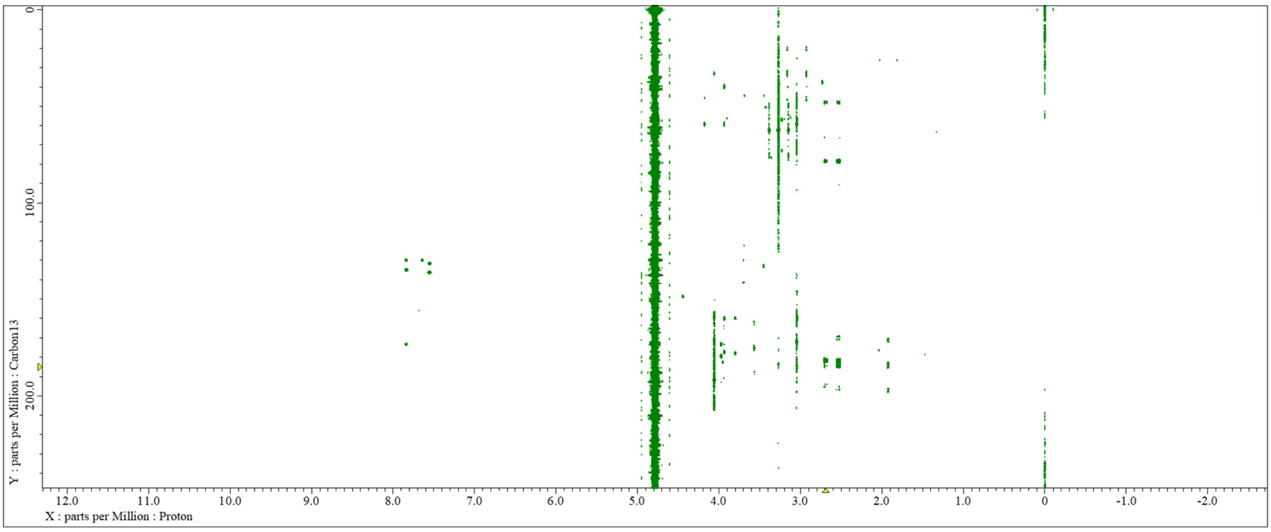

Figure S2. 2D-NMR experiments performed on urine samples. Since no qualitative differences were found in profiles of workers compared to healthy unexposed only spectra obtained from the analysis of one worker's sample are shown as an example.

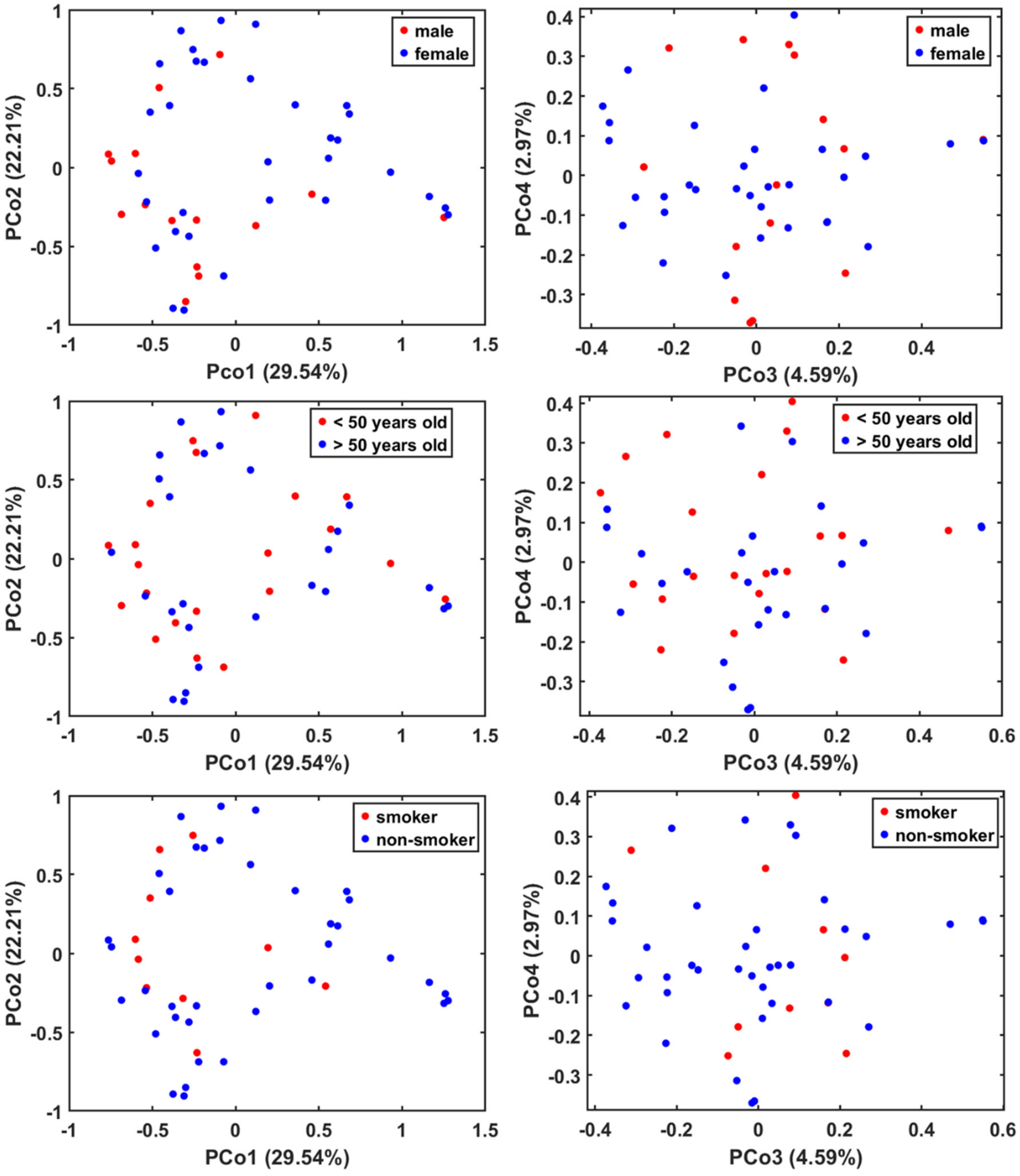

Figure S3. Score plots obtained from Unsupervised Random Forest analysis color-coded based on plausible confounding factors.
